# Changing Medical Paradigm on Inflammatory Eye Disease: Technology and Its Implications for P4 Medicine

**DOI:** 10.3390/jcm11112964

**Published:** 2022-05-24

**Authors:** Takenori Inomata, Jaemyoung Sung

**Affiliations:** 1 Department of Ophthalmology, Juntendo University Graduate School of Medicine, Tokyo 113-0033, Japan; jsung1@usf.edu; 2 Department of Digital Medicine, Juntendo University Graduate School of Medicine, Tokyo 113-0033, Japan; 3 AI Incubation Farm, Juntendo University Graduate School of Medicine, Tokyo 113-0033, Japan; 4 Morsani College of Medicine, University of South Florida, Tampa, FL 33612, USA

Society 5.0 is a novel societal concept proposed by the Japanese government during the 5th Science, Technology, and Innovation Basic Plan [1,2]. Through integrating the physical space and cyberspace, it aims to concurrently promote economic advancements and resolve social problems. Such societal vision inevitably requires a paradigm shift in the field of medicine, calling for the incorporation of comprehensive digital forms of medical big data into clinical practice. Personalized medical information, such as subjective symptoms, biometrics, lifestyle pattern, and environmental information, may be extracted from IoMT (Internet of Medical Things) or sensors attached to commonplace smart devices, which is gaining traction for its potential to add value in healthcare. In addition, community-wide medical and billing data from electronic medical records may add epidemiological insights, and, conversely, genomics or multiomics data may provide multidimensional, molecular-level details on one’s dynamic physiology. With a robust dataset and recent advancements in the information and technology sector, it is becoming increasingly feasible to rapidly analyze medical big data through artificial intelligence (AI) analysis, which appears to have strong implications in adopting core values of P4 (predictive, preventive, personalized and participatory) medicine in future healthcare [2,3]. In this new paradigm, medical big data and its analysis could bring a more accurate community and global health profile, which will lay foundations for a better and tailored predictive models, diagnostic standards, and intervention choices [4]. Furthermore, the digitization of medicine could shift the traditional facility-based, ex-post facto care infrastructure to one that operates within one’s daily life with an emphasis on patient-centered predictive and longitudinal medicine [2,5].

As the aging society and digitalization progresses in the modern era, visual function has become increasingly critical for one’s quality of life (QOL) [6,7]. The eye is one of the few organs that possessed immune privilege to control excessive inflammation and to maintain homeostasis [8,9,10]. Reversely, however, eye tissue frequently experiences irreversible, detrimental visual impairment if such inflammatory changes are to occur [11]. Examples include autoimmune uveitis, keratitis, dry eye disease (DED), allergic conjunctivitis, and corneal allograft rejection, all of which may cause longstanding damage to one’s quality of vision and QOL. The effects of inflammation are often critical in corneal endothelial cells that maintain corneal transparency and in retinal photoreceptor cells, as they do not regenerate in vivo [12,13]. T-cell mediated immune responses play a central role in DED [14], the most prevalent ocular surface disease. However, current mainstay treatment revolves around preventing exacerbations and symptomatic management with minimal routes to reverse the disease course [15,16]. Corneal allograft rejection is also a frequently encountered inflammatory process. Albeit with great response to existing treatment with corticosteroids and immunomodulators [17], the regimen pathway remains singular due to limited pharmacological targets and individualized strategies. With failed management, the only option is repeated corneal transplantation, and rejection rates only increase with repeated, high-risk corneal transplantation to the graft bed [18]. Although their pathophysiology is mainly cell death-driven, inflammatory mediators are increasingly thought to play an important role in retinitis pigmentosa (RP) [19] and other forms of hereditary retinal dystrophies, all of which still do not have a curative option [20]. As numerous ocular diseases involve inflammatory changes to a significant degree, a better understanding of the inflammatory dynamics at a personal level may have strong implications for the prevention of permanent visual impairment and decreased QOL for a wide population.

In the context of any inflammatory disease pathogenesis, a disease rarely develops as a dichotomy of present or absent disease state. Instead, it presents as a disease spectrum with various factors, leading to phenotypes that ranges from subclinical to severe stages [21,22,23]. DED is a highly multifactorial disease, characterized by three major categories of risk factors: environmental, lifestyle, and host factors [21,24,25]. Specific factors include humidity, pollen status, particulate matter 2.5, diet, tobacco use, physical activity, contact lens use, age, sex, and family history, all of which can affect one’s susceptibility, severity, and prognosis. Its presentation varies amongst individuals, often with highly heterogenous sets of symptoms, including eye dryness, decreased visual acuity, eye strain, photophobia, and depressive mood symptoms [25,26,27]. However, despite understanding the complexity of DED pathophysiology, current treatment standards for DED are hardly tailored to target specific factors and maximize treatment efficacy [28,29]. In overcoming this hurdle in pursuing treatment optimization, the healthcare system must first gain a holistic insight into one’s health through understanding their symptoms and related lifestyle data, as well as a disease stratification strategy based on this information to optimize management plans for individuals [26,30,31].

Recently, the field of cross-hierarchical integrative data-driven biological research has received attention owing to the development of medical big data and AI technologies [2,30,31,32]. Such analyses may have implications for elucidating various pathophysiology as they encompass all levels of cellular function from intracellular molecular dynamics to end phenotypes [18,28]. The IoMT and the expanding landscape of biomedical big data allows for the integration of multiomics, patient reported outcomes (PROs) [33], and real-time inputs from now-commonplace sensors attached to mobile devices [29,34,35,36]. A multilevel understanding of a disease pathophysiology could unveil various biomarkers and pharmaceutical targets, possibly enabling targeted medical management for specific ocular inflammatory mediators. Since the discovery of the relatively novel LFA-1 antagonist approved for DED therapy [37], various targets of the T-cell mediated inflammatory cascade have emerged as possible pharmacological targets, including Th17, Th1, TSP-1, and LXA4 [14]. Notably, the shift towards integrative research is particularly promising for hereditary diseases with the involvement of numerous genes such as RP, as its disease process involves a complex cascade of retinal degeneration, oxidative stress, cellular calcium homeostasis, and microglial inflammatory activity [38].

Through proposed changes in Society 5.0 and adoption of mobile health (mHealth) principles, providers and researchers may be able to deduce certain ocular inflammatory dynamics at an individual level with correlating digital phenotypes [39] (i.e., ePROs, sensor data, on-screen time, blink pattern recognition) [5]. Various mHealth-driven approach to gain personalized data have gone underway with significant findings, particularly in chronic disease and mood disorder management [36,40,41]. This expansion of perspective helped elucidate new aspects of pathophysiology, disease course, biomarkers, and disease subgroups [26,30,31], laying ground for tailored therapeutic targets for highly diverse and heterogenous diseases, including various immune-mediated ocular disorders. Additionally, one of the most significant changes to standard care would be the transition away from the traditional facility-oriented care, to one that can provide healthcare within one’s daily life, which may have implications in inflammatory diseases that often require long-term management. The global shift towards digital and physical space integration may allow for non-intrusive longitudinal care, as well as create new value to medicine through truly bringing values of P4 medicine to healthcare.

## Conclusions

Ocular inflammatory diseases are highly multifactorial and heterogenous in its presentation. With the proposed paradigm shift in healthcare under Society 5.0, a hierarchical, cross-sectional analytic approach of medical big data may help elucidate pathophysiology, novel biomarkers, preventative strategies, and new pharmacological agents for complex immune-mediated diseases. These innovations should lay foundations in realizing P4 medicine, guiding healthcare towards patient-centered care.
